# Laparoscopic and endoscopic cooperative surgery for early gastric cancer: Perspective for actual practice

**DOI:** 10.3389/fonc.2022.969628

**Published:** 2022-10-03

**Authors:** Peng-yue Zhao, Zhao-fu Ma, Ya-nan Jiao, Yang Yan, Song-yan Li, Xiao-hui Du

**Affiliations:** Department of General Surgery, First Medical Center of the Chinese People's Liberation Army (PLA) General Hospital, Beijing, China

**Keywords:** early gastric cancer, laparoscopic and endoscopic cooperative surgery, sentinel nodes, endoscopic submucosal dissection, endoscopic resection

## Abstract

Early gastric cancer (EGC) has a desirable prognosis compared with advanced gastric cancer (AGC). The surgical concept of EGC has altered from simply emphasizing radical resection to both radical resection and functional preservation. As the mainstream surgical methods for EGC, both endoscopic resection and laparoscopic resection have certain inherent limitations, while the advent of laparoscopic and endoscopic cooperative surgery (LECS) has overcome these limitations to a considerable extent. LECS not only expands the surgical indications for endoscopic resection, but greatly improves the quality of life (QOL) in EGC patients. This minireview elaborates on the research status of LECS for EGC, from the conception and development of LECS, to the tentative application of LECS in animal experiments, then to case reports and retrospective clinical studies. Finally, the challenges and prospects of LECS in the field of EGC are prospected and expounded, hoping to provide some references for relevant researchers. With the in-depth understanding of minimally invasive technology, LECS remains a promising option in the management of EGC. Carrying out more related multicenter prospective clinical researches is the top priority of promoting the development of this field in the future.

## Introduction

With people’s increasing attention to healthy diet and the popularization of *Helicobacter pylori (Hp)* eradicating treatment, the incidence and mortality of gastric cancer in European and American countries have been significantly reduced in recent years, ranking after the tenth among all tumors (1). However, gastric cancer is still a heavy burden in East Asian countries such as China, Japan and South Korea (2). Noteworthily, early gastric cancer (EGC) has a desirable prognosis compared with advanced gastric cancer (AGC). Statistics from the Japan Gastric Cancer Association show that the five-year disease-specific survival rates of EGC invading the mucosa and submucosa reach 99.3% and 97.2%, respectively (3). Therefore, diagnosing and managing gastric cancer as early as possible is the most cost-effective mean to improve the prognosis of gastric cancer patients.

Currently, endoscopic resection and laparoscopic resection are the mainstream surgical methods for EGC, but both have certain inherent limitations (4). For example, endoscopic resection of EGC has strict indications, and the size, location of the tumor, and whether it is accompanied by ulcers are critical factors that need to be considered. Although laparoscopic resection is guaranteed in terms of radical resection, the increase in postoperative complications seriously affects the quality of life (QOL) in EGC patients (5). Fortunately, the advent of laparoscopic and endoscopic cooperative surgery (LECS) has overcome these limitations to a considerable extent, which not only expands the surgical indications for endoscopic resection, but greatly improves the QOL in EGC patients (6). LECS refers to a new surgical method that combines the advantages of endoscopy and laparoscopy, which was first proposed by Japanese scholar Hiki and applied to the administration of gastrointestinal stromal tumors (GIST). With the rapid development of surgical instruments and the accumulation of surgical experience of surgeons, LECS is gradually applied in other gastric tumors, even EGC.

Although numerous clinical studies and reviews on LECS in gastrointestinal tumors have been published, to the best of our knowledge, a comprehensive study focusing on the application of LECS in EGC has not yet been reported. This minireview elaborates on the research status of LECS for EGC, from the conception and development of LECS, to the tentative application of LECS in animal experiments, then to case reports, retrospective clinical studies and ongoing prospective clinical trials. Finally, the challenges and prospects of LECS in the field of EGC are prospected and expounded, hoping to provide some reference for relevant researchers.

## History of combined endoscopic and laparoscopic surgery

Since the beginning of the 21st century, the rapid development of endoscopic and laparoscopic technology has pushed gastrointestinal surgery into the era of minimally invasive surgery. Meanwhile, the surgical concept of EGC has altered from simply emphasizing radical resection to both radical resection and functional preservation. To solve the thorny issue that laparoscopic technique needs to be appropriately altered according to the location and size of gastric tumors, Hiki et al introduced a novel surgical procedure named LECS, which could be conducted for gastric SMTs resection and unaffected by tumor site and size (6). Nevertheless, the indications of LECS were quite conservative when it was first proposed, and the majority of applicable tumors were benign ones such as gastric submucosal tumors (SMTs) and GIST. In the same year, Japanese scholars Abe et al. successfully performed laparoscopy-assisted endoscopic full-thickness resection (LAEFTR) with lymphadenectomy in a 62-year-old EGC patient (7). This study confirmed that LAEFTR with lymphadenectomy was a minimally invasive and effective option for the treatment of EGC patients, reducing the extent of gastrectomy without compromising curability.

Although the advent of LECS has successfully solved the problem that the location and size of the tumor affect the surgical method, and the applicable indications of LECS have been extended to EGC (8), however, the gastric cavity and the abdominal cavity need be connected during the operation, which will lead to the increasing risk of gastric peritoneal metastases. Facing these challenges, gastroenterologists did not stop innovating and turned their attention to reducing or even avoiding the occurrence of gastric cancer peritoneal metastasis during LECS. In 2011 and 2012, Japanese scholars Goto and Inoue proposed two improved LECS, non-exposed endoscopic wall-inversion surgery (NEWS) (9) and full-layer resection of gastric wall with non-exposure technique (CLEAN-NET) (10), respectively. Unlike LECS, these two surgical operations do not need to open the stomach cavity, thus avoiding the problem of cancer cell metastasis caused by the communication between the stomach cavity and the abdominal cavity.

**Figure 1:**
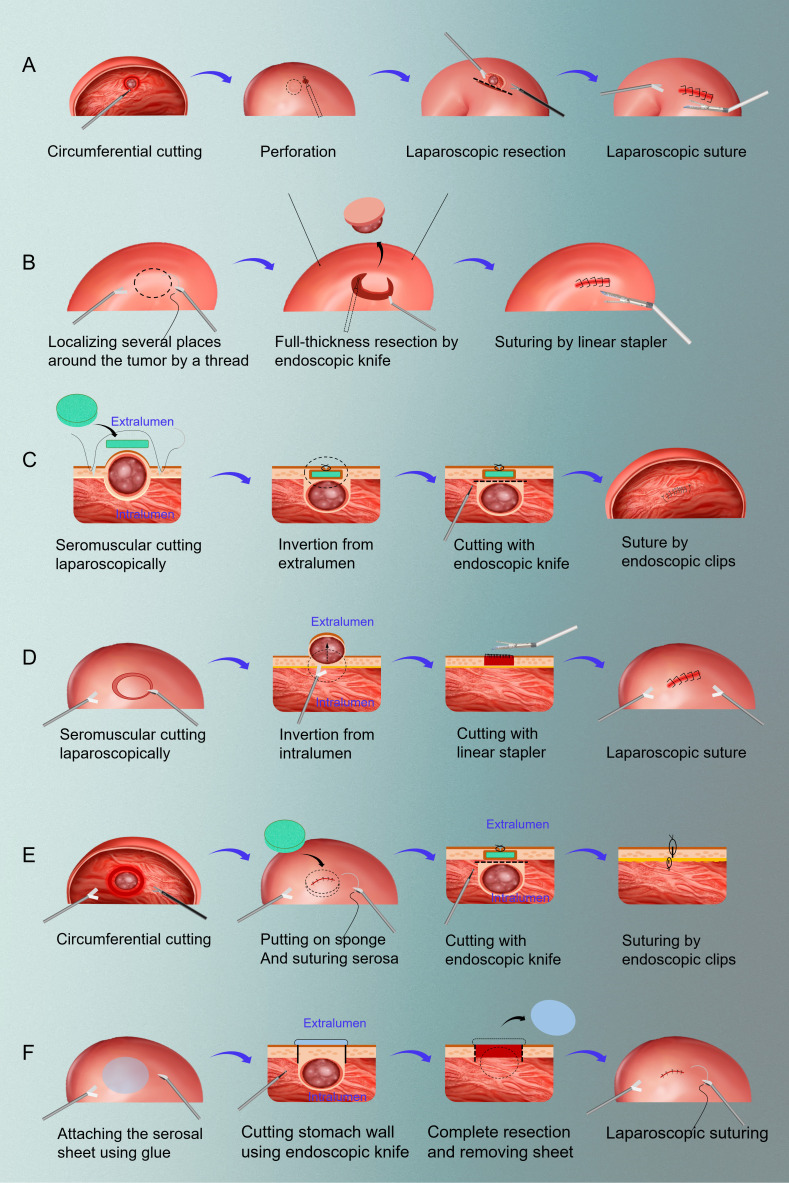
The schemas of six resection methods of LECS. **(A)** classical LECS; **(B)** inverted LECS (Crown Method); **(C)** NEWS; **(D)** CLEAN-NET; **(E)** closed LECS; **(F)** sealed EFTR. LECS, laparoscopic and endoscopic cooperative surgery; NEWS, non-exposure endoscopic wall-inversion surgery; CLEAN-NET, full-layer resection of gastric wall with non-exposure technique; EFTR: endoscopic full-thickness resection.

In the following years, LECS-related research achievements entered a stage of prosperity. NEWS has been gradually applied to clinical patients from initial tentative exploration in pig models (11). Moreover, the application of LECS is no longer limited to gastric tumors, and it has been successfully reported in duodenal tumors and colorectal tumors (12, 13). In 2017, Professor Kikuchi introduced the closed LECS surgical method in English, which was similar to the previous NEWS and CLEAN-NET surgery. In fact, Dr. Nishizaki is the developer of Closed LECS. Closed LECS avoids the opening of the stomach cavity, and the operation is easier and convenient (14). One year later, Takechi et al. successfully applied LECS to advanced gastric cancer for the first time with the informed consent of patient, marking an epoch-making breakthrough in the history of endoscopic laparoscopic combination therapy (15). In 2019, Japanese scholar Kitakata et al. proposed a new improved LECS procedure, sealed full-thickness resection (sealed EFTR), which has been successfully applied in ex vivo and *in vivo* porcine gastric cancer models (16). In conclusion, since the concept of classical LECS was proposed in 2008, surgeons dedicated to the better application of LECS in early gastrointestinal tumors have not stopped the pace of innovation. After more than ten years of development, up to now, there are six kinds of LECS and their improved operation methods, namely, classical LECS, inverted LECS (Crown Method), NEWS, CLEAN-NET, Closed LECS and Sealed EFTR (17). The schemas of these methods can be found in **Figure 1**.

However, we need to be clear about: although LECS and its modified surgical methods have been tentatively applied in EGC, there is great controversy about LECS as a therapeutic method for radical tumor resection. Therefore, LECS for EGC is only applicable in the following cases: 1. cases in which ESD is indicated but technically difficult to perform; 2. resection as a palliative treatment for cases in which standard gastrectomy is dangerous due to advanced age and severe comorbidities; 3. a clinical trial with sentinel node biopsy.

According to the latest Japanese gastric cancer treatment guidelines (the 6th edition), radical gastrectomy plus D_1+_ lymph node dissection is the first choice for the treatment of EGC with lymph node metastasis, while for those without lymph node metastasis, local resection of gastric cancer can be performed by endoscopic or laparoscopic surgery. To more accurately and quickly identify the lymph node metastasis of EGC, the concept of sentinel lymph node (SN) came into being and showed a good clinical prospect. The overall assessment of lymph node metastases in EGC by sentinel lymph nodes has been supported by evidence from numerous retrospective studies. Encouraged by several favorable single-institution reports, Japanese scholar Kitagawa et al. conducted a multicenter, single-arm, phase II study in 2013 to evaluate the safety and efficacy of SN mapping using a standardized dual tracer endoscopic injection technique in gastric cancer (18). Through the analysis of 397 eligible patients, the study found that the accuracy of SN biopsy in the assessment of gastric node metastasis was as high as 99%, and no severe adverse reactions related to endoscopic tracer injection or SN mapping procedures were observed, demonstrating the safety and efficacy of endoscopic dual tracer method for SN biopsy in superficial, relatively small gastric adenocarcinoma.

Although lymph node drainage in gastric cancer is quite complex, studies have confirmed that the probability of regional lymph node metastasis in EGC is not high. Therefore, some experts believe that standard D_2_ radical gastrectomy for patients with EGC is excessive, and lymph node biopsy may improve this embarrassed situation to a certain extent. Noteworthily, LECS combined with SN biopsy has strict indications: clinical T_1_N_0_M_0_ or T_2_N_0_M_0_ with single primary lesions (≤4 cm) without previous treatment, fine general condition and able to tolerate surgery, without a history of drug-related allergy or active asthma etc. Moreover, some limitations of LECS combined with SN biopsy are still inevitable, for example, SN biopsy has a certain percentage of false-negative, the oncological efficacy and QOL improvement of LECS combined with SN biopsy have not been validated and supported by multicenter prospective studies (19).

In 2016, many experts dedicated to gastric cancer research in Korea led the SEntinel Node ORIented Tailored Approach (SENORITA) trial, a multi-center randomized phase III clinical trial comparing laparoscopic gastric-sparing sentinel lymph node dissection and standard gastrectomy plus lymph node dissection for EGC (20). Encouragingly, this study, which published results in 2022, showed that laparoscopic sentinel node navigation surgery (LSNNS) did not demonstrate noninferiority to laparoscopic standard gastrectomy (LSG) in terms of 3-year disease-free survival (3y-DFS), and LSNNS had better long-term QOL and nutrition than LSG (21). Considering that there remain several ongoing randomized controlled trials (RCTs) in Japan that have not reported results, a comprehensive consideration of a multi-country prospective clinical trials would be more convincing.

## Application of LECS for EGC

### Animal experimental studies related to LECS for EGC

EFTR is a commonly used surgical procedure for the treatment of early gastric benign and malignant tumors, but its indications are limited due to the inevitable spread of tumor cells into the abdominal space on account of transmural communication. In view of this, Goto et al. invented a new method for EFTR that does not require transmural communication (NEWS) and explored its feasibility in three ex vivo porcine models (9). The surgical operation of NEWS can be summarized into four steps. First, a flexible endoscope is used to make a mark around the model lesion. Second, a circumferential serous-muscular incision is made externally with electrocautery, based on markers and endoscopic gastric navigation. Third, linearly suture the muscle layer and the lesion medially. Finally, a mucosal-submucosal circumferential incision is made with electrocautery under the endoscope. It is worth mentioning that there was no perforation or obvious air leakage during or after the resection. The authors believe that NEWS is an effective minimally invasive endoluminal procedure for gastric SMTs with or without ulcers, or even node-negative EGCs that are difficult to remove by endoluminal submucosal dissection (ESD). Four years later, goto’s research team conducted a survival study on a live pig model to explore the safety and feasibility of NEWS with sentinel node basin dissection (SNBD) (22). The lesions were completely resected and the mean operative time was 170 minutes (130-253 minutes), and all pigs survived with no undesirable events. All pigs were sacrificed one week later and necropsy showed no signs of serious complications. This animal survival study illustrated that NEWS combined with SNBD was safe and feasible, and may provide minimal local excision for potentially node-positive EGC patients without the risk of tumor spread.

Similarly, Mitsui from Japan and Kim from South Korea have also explored the feasibility of LECS for EGC in animal experimental models, respectively. In the former research, 6 explanted pig stomachs and 6 live pigs were selected and then completed the NEWS operation (23). All 12 lesions were successfully resected without perforation. Three pigs were monitored for 7 days, all survived without adverse events, and necropsy revealed no leaks or abscesses, demonstrating that NEWS is technically feasible and safe in both *in vitro* and *in vivo* pig studies. The latter performed non-exposure endo-laparoscopic full-thickness resection with simple suturing technique (NESS-EFTR) in 4 pigs (24). All pigs underwent complete excision and no adverse events occurred. The mean operation time was 137.0 minutes. Gross and microscopic examination of the excision site showed healing with no evidence of leakage or infection, indicating that NESS-EFTR was feasible in animal models. The summary of animal experimental studies related to EGC and LECS is presented in **Supplementary Table S1**.

### Case reports related to LECS for EGC

After animal experiments demonstrated the feasibility and safety of LECS, surgeons began to try to apply the procedure to clinical patients. Proverbially, compared with endoscopic resection, laparoscopy facilitates intraoperative lymph node dissection and can perform local resection from outside the stomach, thereby ensuring the integrity of lesion resection. In 2008, Japanese professor Abe attempted to utilize LAEFTR to treat a 62-year-old EGC patient. LAEFTR consists of 4 main procedures: first, a circumferential incision deep to the submucosa is made around the lesion by ESD technique; second, an endoscopic incision is made laparoscopically over three-quarters of the circumference of the above submucosal incision; next, complete the remaining quarter-circumferential laparoscopic full-thickness incision in the peritoneal cavity; finally, laparoscopic suturing of the gastric wall defect. The entire laparoscopic procedure was successfully completed with a total of 389 minutes and no adverse events occurred. The entire peripheral tumor of the specimen was negative, 23 lymph nodes were free of cancer cells, and the patient recovered well after surgery and did not affect the postoperative QOL. These results indicate that LAEFTR combined with lymphadenectomy is effective in the treatment of EGC (7).

To achieve proper gastric wall resection, Hiki et al. unveiled the LECS and applied it to the resection of gastric SMTs. In LECS, the site of the tumor is first confirmed by endoscopy, followed by submucosal dissection by endoscopic endoscopy to ascertain the appropriate resection line. Then, the seromuscular layer is laparoscopically dissected, and the incision line is closed with a laparoscopic stapler. Nunobe et al reported a case of laterally diffusing intramucosal gastric cancer with a diameter of 6 cm located in the gastric fornix, and they successfully employed LECS (8). The entire operation time was 152 minutes, and the estimated intraoperative blood loss was 0 ml. Postoperative pathological report confirmed that the tumor was located in the mucosa without lymphatic or venous invasion, and the resection margin was negative. The successful application of LECS in EGC proves that if the EGC meets the criteria for endoscopic resection and there are technical difficulties in performing ESD, it will be a good indication for LECS. The authors also expected that if the concept of sentinel lymph nodes was established, the LECS indications for EGC will be expanded in the future.

As a new minimally invasive surgery that can effectively avoid intraoperative proliferation of gastric cancer cells, NEWS combined with sentinel node navigation surgery can minimize the size of lymph node dissection, whose feasibility has been demonstrated in pig survival studies (9). Goto et al were the first to report a clinical case of NEWS with SNBD for diffuse intramucosal EGC with ulceration in a 55-year-old female patient (11). The operation time was 270 min, and there were no complications. The patient was discharged from the hospital 10 days after the operation and the final pathological diagnosis was consistent with preoperative and intraoperative assessments. This case and Niimi’s study (25) re-validate the feasibility and safety of NEWS plus SNBD, which is expected to be a promising, minimally invasive, function-sparing procedure for potentially node-positive EGC.

Coincidentally, Kato et al. reported the successful treatment of intramucosal differentiated gastric cancer with CLEAN-NET (26). The patient was an 80-year-old man who was diagnosed with intramucosal differentiated gastric cancer by gastroscopy and preoperative pathology. The authors used CLEAN-NET to resect the tumor without any complications under the informed consent of the patient. The postoperative pathological diagnosis was basal gland-type gastric cancer (GAFT) without lymphatic involvement, and the surgical margins were all negative. This case demonstrates that CLEAN-NET is an effective regimen for the treatment of gastric cancer patients with low-risk lymph node metastases, preventing not only the removal of excess gastric wall, but also the exposure of cancer cells to the abdominal cavity. The detailed information of case reports related to LECS for EGC can be seen in **Supplemental Table 2**.

### Clinical studies related to LECS for EGC

There are numerous similar clinical studies, and researchers have demonstrated the feasibility of various LECS methods, such as ESD combined with laparoscopic lymph node dissection (LLND), hybrid natural orifice transluminal endoscopic surgery (NOTES), etc. (27, 28)

CLEAN-NET is a non-exposed tumor technology that combines the characteristics of laparoscopy and endoscopy. Full-thickness resection of gastric tumors is maneuverable when endoscopy is combined with laparoscopy. However, a major handicap of this procedure is that stomach contents may flow into the abdominal cavity through the open stomach wall incision, thereby increasing the risk of tumor dissemination. Therefore, it is urgent to develop a non-exposed technology to effectively solve this drawback. CLEAN-NET is developed using a serous muscle incision that preserves the continuity of the mucosa, which acts as a clean net to block the communication between the gastric and abdominal cavities. The mucosa surrounding the tumor specimen is then stretched by using a serous muscle incision and the raised full-thickness gastric wall is finally sutured. At this time, the tumor is completely exposed outside the gastric cavity, which can be easily removed by laparoscopy. This operation can control the area of gastric resection to a minimum on the premise of ensuring radical resection. Inoue et al performed CLEAN-NET in 24 consecutive patients (16 gastric cancer and 8 GIST), and the procedure was successful without complications (10).

Sentinel lymph node navigation is an emerging surgical technique in recent years, but the clinical efficacy of this technique for local gastrectomy and regional lymphadenectomy remains unclear. Therefore, Hur et al constructed a prospective pilot study to evaluate the efficacy of LAEFTR combined with sentinel lymph node navigation in patients with EGC (29). The study finally included 9 patients and successfully implemented LAEFTR with sentinel node navigation. The mean operation time and postoperative mean hospital stay were 183.3 minutes and 5.9 days, respectively. Abdominal and pelvic computed tomography examinations did not reveal any recurrence at 6 months after surgery.

There remains a body of single-center retrospective clinical studies exploring the treatment of EGC with LECS, but based on the epidemiological characteristics of the high incidence of EGC in Japan, South Korea and other East Asian countries, majority of these studies are led and carried out by scholars from these countries (30, 31). However, it is undeniable that these studies have provided clinical evidence for the development of LECS and made a significant contribution to its popularization. Fortunately, in the past five years, LECS has not only been carried out in Germany, Japan, and South Korea, but also gradually introduced in China, the Czech Republic and other countries in the world (32, 33). The detailed information of studies related to LECS for EGC can be seen in **Table 1**.

**Table 1:** Summary of studies related to LECS for EGC.

Ref.	Year	Country	Number of cases	Surgery	Conclusion
Abe et al (27)	2005	Japan	5	ESD and LLND	This combination treatment was a potential, minimally invasive method, and may obviate unnecessary gastrectomy without compromising curability for EGC patients having the potential risk of LNM.
Cho et al (28)	2011	Korea	14	Hybrid NOTES	Hybrid NOTES could be a bridge between endoscopic resection and laparoscopic surgery and may prevent extensive gastrectomy in patients with EGC
Inoue et al (10)	2012	Japan	16	CLEAN-NET	CLEAN-NET potentially avoided tumor dissemination. CLEAN-NET combined with regional lymph node dissection may suggest further application of this procedure to submucosal cancer.
Hur et al (29)	2014	Korea	9	LAEFTR	This technique could be a novel treatment strategy for gastric cancer patients with inconclusive diagnoses, who would typically undergo laparoscopic gastrectomy or endoscopic resection.
Hajer et al (32)	2018	Czech Republic	2	NEWS	NEWS combined laparoscopic and endoscopic techniques, and preserved the full function of the stomach.
Aoki et al (30)	2018	Japan	7	LECS	LECS was likely to be effective for cases involving intra-mucosal gastric carcinoma that are difficult to treat by ESD due to ulcer scars.
Okubo et al (31)	2020	Japan	25	CLEAN-NET and SNNS	CLEAN-NET with SNNS preserved a better QOL and nutrition status than LADG in patients with early gastric cancer.

LECS, Laparoscopic and endoscopic cooperative surgery; EGC, Early gastric cancer; ESD, Endoscopic submucosal dissection; LLND, Laparoscopic lymph node dissection; LNM, Lymph node metastasis; NOTES, Natural orifice transluminal endoscopic surgery; CLEAN-NET, Full-layer resection of gastric wall with non-exposure technique; LAEFTR, Laparoscopy-assisted endoscopic full-thickness resection; NEWS, Non-exposure endoscopic wall-inversion surgery; SNNS, sentinel node navigation surgery; QOL, Quality of life; LADG, Laparoscopic distal gastrectomy.

## Challenges and prospects

Although significant progress has been made in the application of LECS in the management of EGC, there are still the following areas for improvement. First, the indications of LECS are relatively limited, and laparoscopic radical resection is still the first choice for EGC with lymph node metastasis. Second, LECS requires the tacit cooperation of endoscopists and laparoscopists, and has a long learning curve. There is still a long way to go to further popularize the application of LECS in EGC. Moreover, most of the relevant studies have small sample sizes and are mainly reported in Japan and South Korea, which cannot reflect the global research status. Whether other countries in the world can complete this relatively complex operation remains to be discussed. Finally, most studies related to LECS and EGC are retrospective studies, which may overestimate the real performance of LECS due to selection bias.

Despite the fact that LECS has made major breakthroughs in EGC, we have more expectations for the optimization of LECS. For instance, with the gradual improvement of residents’ health awareness and increasing people take health check-ups, the number of EGC patients will undoubtedly grow. How to expand the applicable indications of LECS and provide more choices for EGC and even AGC patients with individual differences is a meaningful research direction. Furthermore, how to simplify the surgical operation of LECS, thereby shortening the learning cycle is also a problem that needs to be considered. Finally, and most importantly, although there are several ongoing clinical trials, this is far from enough. It is urgent to carry out more multicenter and large-sample clinical research on the application of LECS for EGC, so as to provide a solid theoretical basis for the application and popularization of LECS.

## Conclusion

This minireview first elaborates on the research status of LECS for EGC, from the conception and development of LECS, to the tentative application of LECS in animal experiments, then to case reports and retrospective clinical studies, which enables us to have a comprehensive and in-depth understanding of this filed. Finally, the challenges and prospects of LECS in EGC are prospected and expounded. Noteworthily, LECS makes a significant contribution to the development of minimally invasive technology. At the same time, LECS still has certain limitations need to be overcome or improved. To sum up, LECS remains a promising option in the management of EGC, and conducting more multicenter prospective clinical research related to LECS and EGC is the top priority in the future.

## Author contributions

P-yZ performed the majority of the writing, prepared the figures and tables. Z-fM and Y-nJ prepared the tables and performed the drawing of figures. YY and S-yL edited and revised the manuscript. X-hD designed the study. All authors approved the final version to be published.

## Funding

This work was supported by grants from the National Natural Science Foundation of China (No. 81871317) and Key Project of Military Medical Innovation Program (No. 18CXZ025).

## Conflict of interest

The authors declare that the research was conducted in the absence of any commercial or financial relationships that could be construed as a potential conflict of interest.

## Publisher’s note

All claims expressed in this article are solely those of the authors and do not necessarily represent those of their affiliated organizations, or those of the publisher, the editors and the reviewers. Any product that may be evaluated in this article, or claim that may be made by its manufacturer, is not guaranteed or endorsed by the publisher.

## Abbreviations

EGC, early gastric cancer; AGC, advanced gastric cancer; LECS, laparoscopic and endoscopic cooperative surgery; QOL, quality of life; Hp, Helicobacter pylori; GIST, gastrointestinal stromal tumors; SMTs, submucosal tumors; LAEFTR, laparoscopy-assisted endoscopic full-thickness resection; NEWS, non-exposure endoscopic wall-inversion surgery; CLEAN-NET, full-layer resection of gastric wall with non-exposure technique; EFTR, endoscopic full-thickness resection; SN, sentinel lymph node; SENORITA, SEntinel Node ORIented Tailored Approach; LSNNS, laparoscopic sentinel node navigation surgery; LSG, laparoscopic standard gastrectomy; 3y-DFS, 3-year disease-free survival; ESD, endoscopic submucosal dissection; SNBD, sentinel node basin dissection; NESS-EFTR, non-exposure endo-laparoscopic full-thickness resection with simple suturing technique; GAFT, gastric cancer of fundic gland type LLND, Laparoscopic lymph node dissection; LNM, Lymph node metastasis; NOTES, Natural orifice transluminal endoscopic surgery; LADG, Laparoscopic distal gastrectomy.
