# Supplementary material for: Laparoscopic and endoscopic cooperative surgery for early gastric cancer: Perspective for actual practice
Source: Front Oncol. 2022 Oct 3;12:969628. doi: 10.3389/fonc.2022.969628 (PMC9574332; doi:10.3389/fonc.2022.969628)
Supplement: Supplementary file 1 [file DataSheet_1.pdf]

**Supplemental Table S1 Summary of animal experimental studies related to LECS for EGC.**

| Ref. | Year | Country | Number of cases | Surgery | Conclusion |
|---|---|---|---|---|---|
| Goto et al [9] | 2011 | Japan | 3 (pigs)<br>ex vivo | NEWS | NEWS was thought to be effective as a minimally invasive, and minimal-size endoluminal surgery for gastric submucosal tumors with or without ulceration, or even node-negative early gastric cancer that was difficult to resect by ESD. |
| Mitsui et al [23] | 2013 | Japan | 12 (pigs)<br>6 ex vivo<br>6 in vivo | NEWS | NEWS was technically feasible and safe in both ex vivo and in vivo porcine studies. |
| Goto et al [22] | 2015 | Japan | 10 (pigs)<br>in vivo | NEWS and SNBD | NEWS with SNBD was safe and feasible. It may provide patients with possibly node-positive EGC a minimally-sized local resection and minimally-ranged lymphadenectomy without the risk of tumor dissemination. |
| Kim et al [24] | 2015 | Korea | 4 (pigs)<br>in vivo | NESS-EFTR | NESS-EFTR with simple suturing technique was feasible in an animal model. |

| | | | | | |
|---|---|---|---|---|---|
| Kitakata et al [16] | 2019 | Japan | 10 (pigs)<br>3 ex vivo<br>7 in vivo | Sealed-EFTR | Sealed EFTR is a potentially useful technique for the minimally invasive resection of gastric tumor. |

Abbreviations: LECS: Laparoscopic and endoscopic cooperative surgery; EGC: Early gastric cancer; NEWS: Non-exposure endoscopic wall-inversion surgery; ESD: Endoscopic submucosal dissection; SNBD: Sentinel node basin dissection; NESS-EFTR: Nonexposure endolaparoscopic full-thickness resection with simple suturing technique; EFTR: endoscopic full-thickness resection.

**Supplemental Table S2. Summary of case reports related to LECS for EGC.**

| Ref. | Year | Country | Surgery | Conclusion |
|---|---|---|---|---|
| Abe et al [7] | 2008 | Japan | LAEFTR with lymphadenectomy | LAEFTR with a lymphadenectomy was a minimally invasive and effective treatment for selected patients with EGC. |
| Nunobe et al [8] | 2012 | Japan | LECS | If EGC fits the criteria for endoscopic resection but would present difficulty if performing ESD, this is a good indication for the LECS procedure. |
| Goto et al [11] | 2014 | Japan | NEWS and SNBD | NEWS with SNBD is expected to become a promising, minimally invasive, function-preserving surgery to cure cases of EGC that are possibly node-positive |
| Niimi et al [25] | 2015 | Japan | NEWS | NEWS could be utilized as a novel treatment option for node-negative EGC that is difficult to resect by ESD. |

| | | | | |
|---|---|---|---|---|
| Kato et al [26] | 2015 | Japan | CLEAN-NET | CLEAN-NET could be a therapeutic option for GAFT at low risk of lymph node metastasis because it prevents excess wall defect and exposure of cancer cells into the peritoneal cavity. |

Abbreviations: LECS: Laparoscopic and endoscopic cooperative surgery; EGC: Early gastric cancer; LAEFTR: Laparoscopy-assisted endoscopic full-thickness resection; ESD: Endoscopic submucosal dissection; NEWS: Non-exposure endoscopic wall-inversion surgery; SNBD: Sentinel node basin dissection; CLEAN-NET: Full-layer resection of gastric wall with non-exposure technique; GAFT: Gastric cancer of fundic gland type.
